# The history of cardiac xenotransplantation: early attempts, major advances, and current progress

**DOI:** 10.3389/frtra.2023.1125047

**Published:** 2023-07-10

**Authors:** Nicholas R. Hess, David J. Kaczorowski

**Affiliations:** ^1^ Division of Cardiac Surgery, Department of Cardiothoracic Surgery, University of Pittsburgh School of Medicine, Pittsburgh, PA, United States; ^2^ University of Pittsburgh Medical Center Heart and Vascular Institute, Pittsburgh, PA, United States

**Keywords:** heart–heart transplantation, transplant, pig, genetic engineering, xenotransplant, baboon, history

## Abstract

In light of ongoing shortage of donor organs for transplantation, alternative sources for donor organ sources have been examined to address this supply-demand mismatch. Of these, xenotransplantation, or the transplantation of organs across species, has been considered, with early applications dating back to the 1600s. The purpose of this review is to summarize the early experiences of xenotransplantation, with special focus on heart xenotransplantation. It aims to highlight the important ethical concerns of animal-to-human heart xenotransplantation, identify the key immunological barriers to successful long-term xenograft survival, as well as summarize the progress made in terms of development of pharmacological and genetic engineering strategies to address these barriers. Lastly, we discuss more recent attempts of porcine-to-human heart xenotransplantation, as well as provide some commentary on the current concerns and possible applications for future clinical heart xenotransplantation.

## Introduction: the current problem

Heart transplantation remains the optimal solution for select patients with advanced end-stage heart failure (1). Approximately 4,000–5,000 heart transplants are performed worldwide annually (2). Currently, the number of potential heart transplant recipients greatly outweighs the number of available donors. Although the number of annual transplants has increased in recent years, this number is still primarily limited by the availability of donor organs. To address the growing donor-recipient mismatch, numerous efforts have been focused on both expanding the available human heart donor pool, as well as developing mechanical technologies to aid and/or replace the human heart.

In order to expand the current human donor pool, several methods have been adopted, which are summarized in Table 1. These methods include the use of organs from donors outside of the conventional norms of practice, as well as those with hepatitis C (3–6). In addition to these efforts to expand the current pool, recent perfusion technologies and strategies (7, 8) have re-ignited interest in the use of donation after circulatory death (DCD) hearts, a source that had since long been abandoned in the early years of transplantation, but has shown promising results in recent history (9–11).

**Table 1:** Recent efforts to expand the current human cardiac donor pool.

**Use of extended criteria donors**
Age >50 years
Left ventricular ejection fraction <50%
Predicted ischemic time >4 h
Presence of left ventricular hypertrophy (>1.2 cm)
Presence of coronary artery disease
**Utilization of donors with hepatitis C**
**Donation after circulatory death**
Direct procurement
Thoracoabdominal normothermic regional perfusion

Due to the inconsistency and relative shortage of human donor organs, long-term mechanical heart assistance and replacement have been of great interest. The most successful and widely adopted form of durable mechanical support has been the left ventricular assist device (LVAD). Since early development, these devices have evolved from a para-corporeal design to a fully implantable one (12). LVAD therapy has been shown to not only improve life expectancy, but quality of life in patients with advanced heart failure over medical therapy alone (13, 14). While newer generations of LVADs are able to support patients for longer periods of time with fewer device-related complications (15), they continue to have significant shortcomings. These include increased risk of stroke, gastrointestinal bleeding, dependence on anticoagulation, and risk of infection due to an externalized driveline and power source. Because they are designed to support only the left ventricle, they are not ideal for candidates with concomitant right ventricular failure. Total artificial heart devices have been developed for biventricular support (16, 17); however, past results have been disappointing. A newer generation of total artificial heart systems are currently in development (18), but it remains to be seen if these devices can achieve outcomes comparable to those of replacement with living human heart tissue.

Despite our best efforts to expand human donor organ sources, the number of available organs pale in comparison to the number of patients who may benefit from heart transplantation. In the absence of effective mechanical solutions to total heart replacement, man must look to organ sources beyond those of fellow man, and to perhaps those of fellow beast. If made possible, animal-to-human heart xenotransplantation may offer several advantages over human-to-human allotransplantation. Most importantly, xenotransplantation would provide a consistent, predictable source of donor organs and alleviate the current shortages and selection restraints faced by transplanting centers and waitlisted candidates alike. In addition, an elective xenograft harvest would avoid the harmful effects of brain death on transplanted organs, and organ sources could be regulated insomuch to ensure harvested organs are free from exogenous disease and blood-borne pathogens (19). In this report, we describe the early attempts of non-human-to-human organ and heart xenotransplantation, its major obstacles, and current advances within the field.

## Early xenotransplantation

Xenotransplantation has been conceptualized for at least 300 years, with several attempts over the past few centuries. In the 1600s, Jean-Baptiste Denis, physician to King Louis XIV of France, experimented with xenotransfusion across species. In 1667, he transfused blood from a lamb into a 15-year-old boy with a fever (20). That patient went on to recover, and several xenotransfusions followed in other human recipients. The fourth patient likely experienced the first documented case of a transfusion-related acute hemolytic reaction. The patient died, but it was later determined that his wife had also been poisoning him with arsenic. Nevertheless, both the French and English Parliament banned transfusions shortly thereafter, and this ban remained in place in France for over 200 years.

Early experimental xenotransplantation of various tissues began in the 1800s. Skin from various animals (sheep, cats, chickens, frogs, etc.) was used as free or pedicled skin grafts (21). The true success of these early grafts is not well known, as they may have merely covered and provided protection for skin ulcerations, which ultimately healed under the xenograft. Pig-to-human corneal xenotransplantations were also performed in the 1800s (22), more than 60 years before the first human-to-human corneal allotransplantation in 1905 (23). Few other early transplants were attempted, including the placement of slices of either baboon or chimpanzee testicle into human testicles in order to increase testosterone and reverse the effects of aging in older men (24). Performed by Serge Voronoff in Paris, France, these operations likely had no therapeutic benefit, but were mostly well-tolerated.

Table 2 highlights some of the early clinical solid organ xenotransplantation attempts. The first kidney xenotransplantation attempts in human recipients were performed in the early 1900s. In 1905, Princeteau inserted pieces of rabbit kidney tissue into the kidneys of a child with renal failure. However, the child died 16 days later due to pulmonary complications (25). In 1906, Jaboulay attempted two kidney xenotransplants using pig and goat kidneys using vascular anastomoses. In addition, Unger attempted non-human primate (NHP)-to-human xenotransplantations. Unfortunately, in the transplants performed by both Jaboulay and Unger, the renal xenografts experienced early failure due to vascular thrombosis (25, 26). Short-term success with kidney xenotransplantation would not be achieved until the 1960s. By this time, human-to-human allotransplantation had been performed, first unsuccessfully in the 1930s, and then successfully described in 1954 by Dr. Joseph Murray who performed a living-donor kidney transplant from one twin brother to another (27).

**Table 2:** Early solid organ xenotransplants.

Surgeon	Number of transplants	Source animal	Year(s)	Longest xenograft/recipient survival
Kidney				
Princeteau	1	Rabbit	1905	Kidney non-viable, child died postoperative day 16
Jaboulay	2	1 pig	1906	Early graft failure, graft removed on day 3
		1 goat		
Unger	1	Monkey	1910	Early graft failure
Hitchcock	1	Baboon	1963	Graft failure at 4 days
Reemtsma	6	Chimpanzee	1964	9-month survival of one recipient
Starzl	6	Baboon	1964	49 days
Liver				
Starzl	3	Chimpanzee	1966–1974	14 days
Starzl	2	Baboon	1992–1993	70 days
Heart				
Hardy	1	Chimpanzee	1964	Immediate graft failure
Ross	1	Pig	1968	Immediate failure
Cooley	1	Sheep	1968	Immediate failure
Barnard	2 (heterotopic)	1 baboon	1977	4 days
		1 chimpanzee		
Bailey	1	Baboon	1984	20 days

During the 1960s, the supply of donor organs was low and chronic dialysis was not available. Given these circumstances, Dr. Keith Reemtsma and colleagues at Tulane University looked to non-human primate sources for donor renal grafts. In his series of xenotransplant experiments, he performed a total of six transplant operations, in each case transplanting two kidneys from a chimpanzee into a human (22, 28). In this first series, published in 1964, six human recipients with terminal uremia requiring dialysis received kidneys from chimpanzee donors. The recipients were treated with pre-transplant azathioprine, actinomycin C, and steroids. In these operations, an *en bloc* segment of donor aorta and inferior vena cava containing the origins of the renal arteries/veins were anastomosed to the recipient external iliac artery and vein, respectively. The donor ureters were anastomosed to the recipient bladder. Most of these xenografts failed in 4–8 weeks, but one patient survived for 9 months after renal xenotransplantation. At autopsy, the chimpanzee kidney did not appear to show signs of rejection, and the patient's sudden death was thought to be due to electrolyte derangement, possibly due to high urine output from the renal xenograft (29). A series of six baboon-to-human renal xenotransplants were also performed by Thomas Starzl while at the University of Colorado (30). In this series, the duration of urine excretion from these xenografts was 10–49 days, with the recipients surviving 19–49 days. Unlike that of Reemtsma and colleagues, there were no longer-term survivors. A few other groups from the United States and France attempted NHP-to-human renal transplant, though these series were very small (22, 31).

The first NHP-to-human liver xenotransplants were performed by Thomas Starzl in the 1960s while at the University of Colorado, utilizing livers from chimpanzees. These first attempts were largely unsuccessful, with the longest-surviving recipient living for only 9 days (32, 33). In the 1990s, after moving to the University of Pittsburgh, Dr. Starzl achieved longer-term survival after liver xenotransplantation when he transplanted two baboon livers into human recipients. In this series, one patient survived 70 days (34).

## First cardiac xenotransplantation efforts

**Figure 1:**
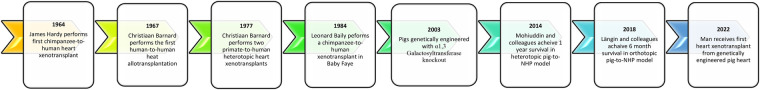
Timeline of significant events in the development of heart xenotransplantation.

James Hardy performed the first NHP-to-human heart xenotransplantation in 1964 at the University of Mississippi Medical Center (35)—3 years before the first human-to-human heart allotransplant described by Christiaan Barnard (Figure 1). Before this first transplant, Dr. Hardy had performed preclinical transplant operations, first in canine models and then in bovine models, exceeding 200 transplant operations before performing his first human transplant. At this time, the ethics of brain death, withdrawal of life support, and organ donation had not been established in the United States or across the world. Dr. Hardy postulated that it may take months to even years until the “perfect” scenario of a hospitalized donor dying of brain damage would align with a simultaneously hospitalized patient dying of terminal myocardial failure at the University Hospital. While awaiting the circumstances for the first human-to-human transplant, a dry run occurred on January 11, 1964. Human-to-human allotransplantation was not performed due to a mistiming of donor and potential recipient circumstances.

Before this “dry run,” Dr. Hardy had spent time in New Orleans with Dr. Reemtsma to witness his previously described NHP-to-human kidney xenotransplantation work. Inspired by this work, he decided to purchase two chimpanzees in anticipation of a chimpanzee-to-human heart xenotransplant, should the specific circumstances needed for human-to-human transplantation not be met for a potential recipient. On 27 January 1964, a 68-year-old man with cardiovascular disease and left leg gangrene, who had been admitted in a comatose state 2 days prior, became the first human heart transplant recipient. The patient was actively dying, supported on vasoactive medications, and without improvement after left leg amputation. Because it was unlikely that a suitable human donor would be available in time, they considered chimpanzee-to-human transplant for this patient. The larger of the two chimpanzees available at the University was selected, and the team proceeded with xenotransplantation. Unfortunately, it became clear that the chimpanzee heart was too small for this human recipient after the xenotransplant was performed, and that this heart could not keep up with the recipient's venous return without significant distention and need for manual cardiac massage and decompression. Ultimately, further support efforts were abandoned, and the patient died. Histopathological assessment determined that antibody-mediated rejection was the primary cause of graft failure (19, 36). The response that followed from both the public and the medical community ultimately deterred Dr. Hardy from attempting heart transplantation again. The first human-to-human heart transplant was then performed in 1967 by Christiaan Barnard in Cape Town, South Africa (37).

Dr. Barnard himself also performed two heterotopic heart xenotransplantations in 1977 (38). The first recipient was a 25-year-old woman who underwent prior aortic valve replacement for severe aortic stenosis. Unfortunately, she experienced hemolysis from the prosthetic Björk–Shiley valve, and she underwent reoperation with aortoventriculoplasty and the insertion of a larger valve. After the reoperation, the patient experienced post-cardiotomy shock and the inability to separate from cardiopulmonary bypass despite the addition of intra-aortic balloon pump. As a salvage maneuver, and in the absence of a suitable donor, Dr. Barnard performed a heterotopic heart transplant using a baboon heart. The patient was able to separate from bypass and was transferred to the intensive care unit. However, within a few hours, the recipient heart, followed by the baboon donor heart, both developed ventricular fibrillation and were not able to be resuscitated after multiple efforts. Dr. Barnard later addended this first report to describe a second heterotopic xenotransplant that was performed on October 13, 1977, using a chimpanzee heart. The recipient was a 60-year-old man who experienced cardiogenic shock and the inability to wean from mechanical support after an aortic valve replacement. After heterotopic implantation of the chimpanzee heart, this patient was weaned from cardiopulmonary bypass and survived 4 days. The heterotopic xenograft experienced a gradual decline in function over those 4 days. A postmortem examination revealed evidence of severe rejection (38).

The next primate orthotopic heart xenotransplantation into a human recipient would later be performed on October 26, 1984, by Leonard Bailey at Loma Linda University (39). At this time, donor infant hearts for transplantation were scarce. This recipient, known as Baby Faye, was a 2.5 kg female infant born at 36 weeks. She was diagnosed with hypoplastic left heart syndrome, which, at the time, carried a grave prognosis. The donor for this operation was a female baboon. Baby Faye was blood type O, Rh positive, which very rarely occurs in the baboon species (3 of 1,307 baboons tested in a prior report) (40). During the operation, the neonate was found to have mitral and aortic valve atresia, as well as a hypoplastic aortic arch. In addition to orthotopic heart transplantation, Dr. Bailey reconstructed the recipient's aortic arch under hypothermic circulatory arrest. After the xenotransplant, Baby Faye was extubated by post-transplant day 3 and was documented to have normal-appearing neurologic function and urine output. She had received an infusion of cyclosporine 38 h before the transplant, and this continued in the post-transplant period, and eventually transitioned to an oral regimen. However, by post-transplant day 14, functional impairment of the xenograft was documented, with later clinical decline and evidence of acute graft rejection by post-transplant day 17. Anti-rejection therapy consisting of antithymocyte globulin, azathioprine, and methylprednisolone was initiated, but unfortunately the patient's clinical status continued to deteriorate with accelerated deterioration in the graft's function. She died later that evening on post-transplant day 20. It was postulated that blood type incompatibility between the donor and recipient may have also contributed to the rejection of the donor xenograft.

In addition to early heart xenotransplantation attempts using NHP hearts, several experiments were also performed using the hearts of non-primate animals (19). Before Christiaan Barnard's heterotopic xenotransplant, in 1968 Donald Ross (London, United Kingdom) performed a heterotopic heart xenotransplant in a 48-year-old man utilizing a pig as a donor animal (41). The xenograft experienced immediate hyperacute rejection and failed within 4 min. In another attempt, he perfused a pig heart with an extracorporeal circuit primed with human blood. This graft also failed immediately and was never transplanted. Also in 1968, Denton Cooley attempted an orthotopic implantation of a sheep heart into a 48-year-old man with ischemic cardiomyopathy in Houston, Texas (42). The heart xenograft unfortunately experienced hyperacute rejection and failed in the operating room. Two other heart xenotransplants were performed in the 1990s in Sasnoweic, Poland, and Sonapur, India, using pig heart xenografts. Though little documentation exists of these transplants, they were reported to function for 23 h and 7 days, respectively (41). Alas, it became clear that significant immunologic barriers existed between humans and other animals, especially in non-primate species.

## Choosing the ideal animal donor

The ideal animal donor for heart xenotransplantation must possess several important features. First, the donor animal must be of adequate size with a heart that can support human circulation. In addition, it should have a heart that anatomically resembles that of the human heart, as well as one that is immunologically compatible. For widespread adoption and utilization of these NHP hearts, other factors should be considered, such as the ease of animal breeding and cost of care, reproductive potential of the animal, and the age at which the animal is mature enough for heart donation. Other factors, such as the risk of xenozoonosis or the spread of diseases across species, must also be taken into consideration. Baboons, as well as other NHPs, share anatomic and immunologic similarities to humans, and for these reasons, NHPs were utilized in the first attempts at animal-to-human heart xenotransplantation. Because of several limitations, including cost and ethical concerns, widespread utilization of baboons as organ source animals is problematic. Although the pig possesses a greater degree of immunological barriers for donation into human recipients (discussed in the next section), commercial breeding for organ donation would prove to be far more cost-effective and less likely to garner ethical scrutiny (22). For these reasons, pigs have been identified as a possible source of organs for xenotransplantation. The advantages and disadvantages of using pigs as source animals for donor hearts are listed in Table 3. In the following section, we describe the major obstacles and advances in xenotransplantation utilizing pigs as a source of organs for transplantation into humans.

**Table 3:** Advantages and disadvantages of heart xenotransplantation with pig source animal over non-human primate.

Advantages	Disadvantages
• Low breeding and raising costs • Higher reproductive potential • Reproductive and donor maturity at younger age • Greater experience with genetic modification/engineering • Lower xenozoonosis risk/ability to raise animals that are “pathogen-free” • Less ethical constraints	• Less anatomically similar to human than NHPs • Greater degree of immunological barriers to overcome with genetic modification/immunosuppression

NHPs, non-human primates.

## Overcoming obstacles to xenotransplantation

In order for porcine-to-human xenotransplantation to be successful, a number of immunological barriers and molecular obstacles must be overcome in order for the pig heart to survive in a human body (43). Below are brief summaries of some of these major barriers, as well as some of scientific advances, which can also be found in Table 4.

**Table 4:** Genetic modifications of pigs used in preclinical xenotransplant models.

Genetic modification	Effect
Hyperacute/acute humoral rejection	
*α*1,3-galatosyltransferase knockout	Prevention of galactose- *α*1,3-galatose (Gal) synthesis and expression
Cytidine monophosphate-*N*-acetylneuraminic acid hydroxylase knockout	Prevention of N-glycolylneuraminic acid (Neu5GC) synthesis and expression
*β*-1,4-acetyl-galactosaminyltransferase 2 knockout	Prevention of Sda synthesis and expression
Inflammation/complement/coagulation dysregulation	
Human thrombomodulin expression	Cofactor in thrombin-induced activation of protein C in anticoagulant pathway
Human endothelial protein C receptor	Membrane protein that enhances activation of protein C in anticoagulant pathway
Human membrane cofactor protein (CD46) expression	Complement regulatory protein
Human decay-accelerating factor (CD55) expression	Complement regulatory protein
Human protein CD47 expression	Reduced recruitment of T cells, neutrophils, and monocytes in areas of inflammation
Heme oxygenase-1 expression	Protection from oxidant-mediated tissue injury
Human A20	Inhibit tumor necrosis factor mediated apoptosis
Cellular rejection and chronic xenograft vasculopathy	
CTLA4-Ig expression	Inhibit T-cell-dependent antibody responses
Major Histocompatibility Complex knockouts	Surface proteins essential for the adaptive immune system
Myocardial overgrowth	
Growth hormone receptor knockout	Transmembrane receptor for growth hormone. Knockout reduces myocardial overgrowth of cardiac xenograft

### Hyperacute rejection and acute humoral xenograft rejection

As was observed in Donald Ross's early first attempt at pig-to-human xenotransplantation, pig xenografts, when perfused with human blood, develop hyperacute rejection similar to that of human-to-human allotransplants performed across blood types. This is in part because humans and NHPs possess pre-formed anti-pig antibodies that bind to the pig vascular endothelium. This binding triggers the complement cascade, which leads to thrombosis, interstitial hemorrhage, and ultimately early failure of the pig xenograft (44, 45). A surface carbohydrate antigen on the pig endothelium, galactose-*α*1,3-galatose (Gal) is the predominant target of these pre-formed antibodies (46). This carbohydrate is lacking in humans and Old World NHPs, but present in most other mammals. In humans and Old World NHPs, this anti-pig (anti-Gal) antibody is believed to develop within the first few months as a response to the colonization of the gastrointestinal tract with microorganisms that express this surface antigen (47). Initial efforts to address the presence of these antibodies were to deplete the recipient of anti-Gal antibodies through plasmapheresis, immunoadsorption, or other methods (19). In addition, another approach was to deplete or inhibit the recipient's complement system (48). Instead of instant graft failure, xenografts could survive days or weeks until the recipient resumed production of anti-Gal antibodies and/or complement. The histological appearance of this “acute humoral xenograft rejection” was similar to that of those that hyperacute rejection.

Genetic engineering has since allowed for a more durable solution to xenograft hyperacute rejection and acute humoral xenograft rejection. By 2003, the production of pigs deficient of this carbohydrate had been made possible through disruption and an enzyme crucial to its synthesis (*α*1,3-galatosyltransferase) (49–51). By blocking the expression of this enzyme, and ultimately the carbohydrate antigen, pig organs transplanted into NHPs demonstrated a dramatic decrease in the incidence of hyperacute rejection (52, 53). In addition, pigs were also engineered to express human complement regulatory proteins (i.e., CD46 = membrane cofactor protein; CD55 = decay-accelerating factor; and CD59 = membrane attack complex inhibitor protein), which would provide protection from the NHP recipient's innate complement system (54). Simultaneous Gal transferase knockout and expression of complement regulatory protein, CD55, further protected pig xenografts from hyperacute rejection and complement activation that may occur in response to other antigens other than Gal (55). In addition to Gal, other carbohydrate pig xenoantigens have been discovered. These include N-glycolylneuraminic acid (Neu5GC) and Sda. While it is possible many humans do not have antibodies to pig cells with triple-knockout of the expression of these three surface antigens (56), baboons may still express additional anti-pig antibodies (57), highlighting the limitations of a baboon xenotransplant recipient model.

To further combat antibody-mediated rejection, pre-transplant depletion of B cells through the use of an anti-CD20 antibody, as well as the suppression of the CD154 (CD40 Ligand or CD40l)-mediated B-cell activation have been effective at prolonging xenograft survival (58–60). The initial attempts at blocking the CD40-CD154 (CD40 Ligand) costimulatory pathway were complicated by thrombocytopenia and thrombotic events, as CD154 is also expressed on platelets (61, 62). However, inhibition of the CD40-CD154 (CD40 Ligand) costimulatory pathway through the use of a primatized (mouse-rhesus chimeric) anti-CD40 antibody (2C10R4) that was not associated with consumptive coagulopathy resulted in the prolonged survival of genetically engineered pig cardiac xenografts in a heterotopic model (58).

### Inflammation and dysregulated coagulation

Transplantation of a pig heart into a NHP results in a significant inflammatory response in the host animal. This response is associated with increased levels of tumor necrosis factor-alpha in the recipient animal (63–66), perhaps related to ischemia-reperfusion injury (67, 68). Despite the use of genetically engineered pigs with knockout of Gal and expression of human complement regulatory proteins, additional thrombotic phenomena have been noted in xenografts transplanted into NHP recipients. Both thrombotic microangiopathy and a systemic consumptive coagulopathy have been observed (69). While not fully understood, this coagulation dysregulation may be, at least in part, due to this state of increased inflammation as well as the recipient animal's innate immunity (70). This hypercoagulable state often cannot be mitigated by either the pig or NHP's endogenous anticoagulant mechanisms, ultimately resulting in thrombotic microangiography, graft dysfunction, and eventual failure. To reduce these thrombotic sequelae, two mechanisms have been proposed: (1) transgenic expression of anticoagulant factors and pharmacological anticoagulation; and (2) a reduction of the inflammatory response.

To reduce the propensity of dysregulated coagulation in pig xenografts, transgenic expression of human coagulation regulation proteins, such as thrombomodulin (71–74), endothelial protein C receptor (75), tissue factor pathway inhibitor (76), and CD39 (77), have been pursued. To reduce the inflammatory state associated with xenotransplantation, etanercept, an anti-tumor necrosis factor-alpha agent, has been administered as an anti-inflammatory medication (78, 79). In addition, genetically engineered pigs that express human inflammatory regulatory proteins, such as heme oxygenase-1 (HO-1) and human A20, have been developed (80, 81).

### Cellular xenograft rejection and chronic xenograft vasculopathy

Unlike human-to-human heart allotransplantation, acute cellular rejection has not been a significant cause of xenograft failure after porcine-to-NHP xenotransplantation. This is likely because humoral, or antibody-mediated, rejection presents such a significant barrier to xenotransplantation, and its rapid development likely results in graft failure before cellular rejection can occur. Nonetheless, as methods to prevent hyperacute and acute humoral xenograft rejection improve, prevention of acute cellular rejection, and ultimately chronic xenograft vasculopathy, will be required to ensure the long-term functionality of transplanted xenografts (43). Immunosuppressive medications commonly used in human allotransplantation may delay graft failure in heart xenotransplantation models but may be coupled with a higher risk of infectious complications (82, 83). Another method of inhibition of the adaptive immune system is through a blockade of T-cell costimulatory pathways. In attempts to block T-cell co-stimulation, genetic engineering of transgenic pigs has been developed that expresses the T-cell co-stimulation blockage agent, CTLA4-Ig (84, 85). Unfortunately, transgenic expression of CTLA4-Ig limited survival in these genetically engineered pigs due to its powerful immunocompromising effects (85). Other areas of areas of interest include genetically engineering pigs with Major Histocompatibility Complex class I knockouts (86).

Chronic xenograft vasculopathy has been documented in some pig-to-baboon xenografts that have survived longer than 3 months (87). The histological appearance is similar to that of chronic allograft vasculopathy seen after human-to-human allotransplantation, characterized by concentric intimal hyperplasia of the coronary arteries and longitudinal, diffuse luminal narrowing of the vessels. However, in xenografts with Gal knockout and expression of human CD46 and thrombomodulin, one surviving more than 2 years, this xenograft vasculopathy was not noted (88). Further study is needed to address the true incidence and impacts of chronic rejection in xenotransplant models.

## Long-term preclinical xenograft survival

Currently, the pig-to-NHP xenotransplantation model remains the standard preclinical model to precede eventual clinical xenotransplantation (89). Similar to that of the first pig-to-human xenotransplant attempts using non-genetically modified pig hearts, the first attempts at pig-to-NHP heart xenotransplantation resulted in very early graft failure. With the addition of some of the genetic modifications of porcine hearts described above, along with improvements in immunosuppression regimens, xenograft survival has been greatly improved.

Early pig-to-NHP heart xenotransplant models consisted of xenografts that were implanted in the heterotopic (non-life supporting) position within the abdomen. In early studies, expression of human complement regulatory proteins was able to extend xenograft survival to several weeks in the heterotopic position (19, 90) as opposed to minutes with unaltered, wild-type pig hearts. With the introduction of Gal knockout genetic engineering, xenograft survival had been improved to almost 6 months in some recipients (91). However, xenografts with knockout of Gal and expression of complement regulatory proteins still experienced thrombotic complications, particularly thrombotic microangiopathy. In response to the problem of ongoing coagulation dysregulation, Mohiuddin et al. evaluated the addition of human coagulation regulatory protein expression (i.e., thrombomodulin) to Gal knockout and complement regulatory protein expression. In this model, with this combination of genetic manipulations and blockade of the CD40-CD40l pathway, they were able to extend heterotopic xenograft survival further, with one graft surviving for 945 days (71, 72). Following the success of porcine-to-NHP xenograft survival in the heterotopic position, researchers now aimed to achieve consistent xenograft survival in the orthotopic, life-sustaining position in order to pave the way toward clinical xenotransplantation.

In the year 2000, the International Society of Heart and Lung Transplantation set a goal for a minimum survival of 3 months to be achieved in 60% of orthotopic, life-sustaining pig-to-NHP xenografts before a clinical xenotransplantation trial could be considered (92). They recommended that at least 10 animals reach this survival point in the absence of life-threatening complications from the genetic modifications and immunosuppression. In 2018, Längin et al. published their success in orthotopic pig-to-NHP heart xenotransplantation (79). In this series, pig hearts with Gal knockout and expression of the complement and coagulation regulatory proteins, CD46 and thrombomodulin, were used. In a first group of five animal transplants, the porcine xenograft was preserved from the time of procurement to the time of implant with a clinically utilized crystalloid preservation solution. However, xenograft survival was disappointing. In a second cohort of four xenotransplant animals, the porcine xenograft was preserved *via* hypothermic (8°C), non-ischemic, continuous perfusion using Steen solution (93), an oxygenated albumin-containing hyperoncotic cardioplegic solution containing recipient animal blood. In this group, one animal was sacrificed due to complications stemming from technical failure, but the other three grafts survived 18, 27, and 40 days, respectively. The authors noted progressive left ventricular stiffening and diastolic failure in these grafts. A histological examination revealed myocardial cell hypertrophy, along with multifocal myocardial necroses, thromboses, and immune cell infiltration. Based on prior preclinical and clinical work and findings (94–97), the team performed a series of five xenotransplants but with the utilization of antihypertensive therapy, mTOR inhibition using temsirolimus, as well as early tapering of steroid medications. One animal died of surgical complications at 51 days, and two others were euthanized at 30 days according to the study endpoint. However, the study endpoint was extended to 6 months for the remaining two animals. At 175 days and 161 days for these animals, respectively, there was no evidence of systolic or diastolic dysfunction, and temsirolimus was discontinued at this point. After discontinuation, cardiac overgrowth and diastolic failure were observed, and the animals survived to 195 and 182 days, respectively. In this last series, multiple variables had been altered. Specifically, the authors added temsirolimus but also weaned steroids early and aggressively controlled recipient blood pressure. Given that multiple changes were made in the experimental protocol, it is not known whether the addition of temsirolimus alone was responsible to suppression of myocardial overgrowth or whether this was due to the other changes that were made in the protocol. Further, it is also unclear whether the hearts eventually became thick after temsirolimus was withdrawn as a result of overgrowth or whether this was due to rejection after temsirolimus was withheld. In one of the animals that survived for 6 months, there was evidence of myocardial necrosis without cellular infiltrates or microangiopathy. The etiology of this myocardial injury is unclear but may represent an undefined immune insult that will require further characterization.

A series of experiments at the University of Maryland confirmed that 6-month survival could be achieved in a life-sustaining pig-to-NHP orthotopic model using genetically engineered cardiac xenografts and a C40 blockade (78). Additional knockout of the growth hormone receptor gene proved to be effective in preventing xenograft overgrowth and obviate the need for temsirolimus (98). Furthermore, Cleveland et al. (99) from the University of Alabama at Birmingham published a series of four pig-to-NHP orthotopic heart xenotransplant experiments in 2022. In all five experiments, donor pigs had Gal knockout. Two donor pigs additionally expressed CD55, and two other pigs expressed CD46 and human thrombomodulin. While two recipient baboons experienced early death, another two baboons survived a total of 90 days and 241 days, respectively. A pathologic examination of the longest-surviving xenograft indicated a mixed antibody-mediated and cellular rejection pattern. All together, these experiments set the stage for initial attempts of transplanting genetically engineered pig xenografts into humans.

## Recent heart xenotransplantation in human recipients

### Pig-to-human xenotransplant at the university of Maryland

On January 7, 2022, a 57-year-old man received a heart xenotransplant from a genetically modified pig at the University of Maryland (100). The recipient had a history of non-ischemic cardiomyopathy, prior mitral valve repair, and presentation with acute on chronic decompensated heart failure. He was hospitalized and supported with intravenous inotrope medications as well as intra-aortic balloon counter-pulsation. Despite this, he continued to have ventricular arrhythmias and cardiac arrests requiring resuscitation and venoarterial extracorporeal membrane oxygenation support. Because of his biventricular failure and history of noncompliance, he was not considered a candidate for left ventricular assist device or human heart allotransplantation. After approval from the U.S. Food and Drug Agency for the experimental transplant, as well as approval from the hospital ethics committee, the operation was performed. A 110 kg pig with 10 gene edits (glactose-alpha-1,3-galactose knockout, Sda blood group antigen knockout, N-glycolylneuraminic acid knockout, growth hormone receptor knockout, human CD46 expression, human decay-accelerating factor (CD55) expression, human thrombomodulin expression, human endothelial cell protein C receptor expression, human protein CD47 expression, and human heme oxygenase 1 expression) was used as the donor heart xenograft. Despite the fact that the operation was complicated by an intraoperative type A dissection, excellent early graft function was observed.

After the transplant operation, the recipient experienced renal failure, requiring continuous renal replacement therapy. He was extubated on postoperative day 2 and venoarterial extracorporeal membrane oxygenation was discontinued on day 4. He was taken to the operating room for an exploratory laparotomy on day 12 for concern for bowel ischemia. Purulent fluid was drained, but there was no evidence of ongoing ischemia or bowel necrosis. Endomyocardial biopsies were taken on post-transplant day 34 without evidence of rejection. On day 43, the patient became more somnolent and hypotensive, and he was re-intubated. The patient was experiencing hypogammaglobulinemia and had worsening lung infiltrates on chest radiography. Due to concern for an infectious process, antimicrobial coverage was broadened and intravenous immune globulin was administered. Weekly serologic testing revealed a gradual increase in pig cytomegalovirus (pCMV) levels (testing of the donor pig's spleen confirmed the animal had been positive for pCMV), which further raised concern for an infectious etiology. By day 49, there was evidence of xenograft failure. The patient became hypotensive with rising serum lactate levels and reduced mixed venous oxygen saturations (33%). Echocardiography revealed preserved ejection fraction, but with significantly increased left and right ventricular wall thickness. He was re-cannulated for peripheral venoarterial extracorporeal membrane oxygenation. After the inability to wean for extracorporeal support, the decision of the transplant team and family was to withdraw support, and the patient died on post-transplant day 60. An autopsy of the heart revealed significant edema with an almost doubling in the xenograft's weight from the time of transplant. Typical patterns of acute cellular or antibody-mediated rejection were not seen, though focal capillary injury in the absence of significant complement deposition was noted—a pattern not typically seen in human-to-human allotransplantation.

The outcome of this xenotransplantation attempt was certainly disappointing, drawing considerable criticism and speculation as to why the xenograft ultimately failed and the patient died. First, the recipient was certainly not an “ideal” operative candidate for transplantation, allo- or xeno-. He had been supported with extracorporeal support for 46 days before transplantation, which was mostly spent bed-bound. Given this, there was much concern about his strength, functional status, and ability to tolerate the operation. Furthermore, his previously established medical noncompliance had eliminated him for consideration for human-to-human allotransplantation. Had the xenograft not failed in this recipient, there is the question of whether he may have survived the postoperative course given his medical comorbidity and frailty. And if he should have survived this perioperative period, it remains to be seen whether later complications pertaining to immunosuppression regimen compliance, which, in terms of xenotransplantation are as complicated if not more than immunosuppression regimens used in allotransplantation, may have developed. Another major concern was the potential consequence of the development of a zoonotic infection in a patient with a history of medical noncompliance. Should a zoonotic infection occur, failure to comply with treatment may allow spread and thereby put others at risk. This should be considered in future attempts at xenotransplantation in humans.

Another important consideration to make was the choice of post-transplant anticoagulation. In this case, the recipient was maintained on an intravenous heparin infusion for anticoagulation, reflecting that of the preclinical work done at the University of Maryland (78). While the necessity of continuous heparin may be debatable, the plan was to keep the post-transplant immunosuppression and medication regimen as similar as possible to preclinical models. Certainly, a heparin infusion would be nearly impossible to maintain in the outpatient setting, especially with a potentially non-compliant patient. It is important to consider whether anticoagulation is necessary, and if so, whether transition to an oral anticoagulant, such as warfarin, or another direct oral anticoagulant. Nevertheless, due to these concerns, it is reasonable to conclude this recipient was not the optimal recipient to demonstrate long-term success in pig-to-human heart xenotransplantation.

The “optimal” pig-to-human xenotransplant recipient is debated. This recipient would have a set of characteristics that would be favorable for tolerating a large operation, but also maintain considerable equipoise among other surgical options for heart failure available to the recipient. Ideally, the recipient would be in good physical condition to tolerate a transplant operation, as well as not have any major contraindications to surgery or immunosuppression. As such, it is no surprise that the “perfect” adult candidate for xenotransplantation would also be an ideal candidate for allotransplantation. With the current status of preclinical work and with the longest-surviving pig-to-NHP models living less than 1 year, it is not reasonable to conclude there is sufficient equipoise between xenotransplantation and allotransplantation. Therefore, the first clinical trials of xenotransplants are not likely to involve healthy adult recipients who would otherwise be suitable candidates for heart allotransplantation. The potential candidates for early trials in cardiac xenotransplantation may include patients with cardiac failure who cannot qualify for allotransplantation or a durable mechanical support strategy (101, 102).

Based on their preclinical work, Cleveland et al. (99) have advocated for the consideration of xenotransplantation in infant populations. As noted in their review, infant heart donors are scarce, attributing to a high rate of mortality among waitlisted candidates aged less than 1 year and with congenital heart disease (e.g., hypoplastic heart syndrome). In fact, they mention that nearly one-third of infants evaluated for heart transplants are denied listing, die while waitlisted, or are removed due to clinical deterioration. The authors conclude that this population may be a potential target of early clinical trials of xenotransplantation in humans. In this scenario, the xenograft would serve as a bridge to human-to-human allotransplantation, given how several pig-to-NHP cardiac xenografts survive to 6 months in NHP models at the University Hospital of Munich, University of Maryland, and University of Alabama at Birmingham. Residual questions that remain to be addressed before a clinical trial include the recipient's immunological and physiological response to allotransplantation after previous xenotransplantation. Early perioperative mortality for patients undergoing surgical management of single ventricle physiology and long-term survival has improved substantially. The use of cardiac xenografts for these patients will need to be weighed against this for patients who are candidates for the Fontan procedure. Those patients at high risk for interstage mortality during single ventricle palliation may be most likely to benefit from cardiac xenotransplantation (103–106).

In addition to recipient candidacy after the University of Maryland case, other factors have been discussed pertaining to the cause of ultimate graft failure and the recipient's eventual death. First, the recipient was found to have rising levels of pCMV (first detected on post-transplant day 20). This is despite husbandry practices used to prevent the passage of pCMV from the mother pig to its offspring as well as rearing of the donor pig in a biosecure facility (100). It is possible that pCMV infection may have contributed, at least in part, to the xenograft failure (107). Another possible contributing factor was the administration of intravenous immunoglobulin to the recipient, both on post-transplant day 43 and again on day 50. As pointed out by Cooper et al. (107) the impacts of IVIg use in preclinical models remain debated, as prior preclinical work has suggested conflicting results as to its benefits and/or harm. Yamamoto and colleagues, based on their prior investigation of five different commercially-available IVIg formulations (108), did not find cytotoxicity of the IVIg preparations to red blood cells from triple-knockout pigs, suggesting its use would be safe in recipients of xenografts from these pigs. However, it is not known whether the IVIg preparations were screened beforehand. Lastly, it is possible the Maryland xenograft failed due to either insufficient immunosuppression or further pig-to-human immunological barriers that have yet to be elucidated and overcome.

### Pig-to-human cadaver xenotransplants at New York University

In June and July of 2022, Dr. Nader Moazami at the New York University Lagone Health Medical Center transplanted two pig hearts into brain-dead human cadavers. These cadavers were supported for 72 h after the transplant. The results of these two experimental xenotransplantations are awaiting publication at the time this article was written (109).

## Conclusions

The successful long-term xenotransplantation of animal hearts into human recipients has been a long sought-after goal with the intent of addressing the shortage of donor hearts that currently limits the number of cardiac transplants in an ever-growing population with heart failure. Since its early conception and attempts dating back to the 1960s, numerous ground-breaking preclinical advances have been made in identifying and overcoming the major immunological barriers to xenotransplantation. Although xenograft survival has greatly improved through the developments of genetic modifications of porcine organ sources and specialized immunosuppression regimens, further study will be required to achieve outcomes comparable to human-to-human allotransplantation. As such, early clinical applications of heart xenotransplantation are likely to not serve as a replacement to human allotransplantation, but rather as a bridge to human allotransplantation in select patients. However, as progress in immunological research continues to move forward and these barriers are overcome, the dream of a nearly endless organ supply for long-term usage may eventually become reality.
